# Highly pathogenic avian influenza virus H5N1 infection in stranded porpoises and dolphins in Chile

**DOI:** 10.3389/fvets.2026.1969182

**Published:** 2026-10-20

**Authors:** Mauricio Ulloa, Víctor Neira, Eva Sierra, Ana Colom-Rivero, Gabriela Muñoz, Magdalena Johow, Felipe Lillo, Daniela Rojas, René Ortega, Raúl Alegría-Morán, Antonio Fernández

**Affiliations:** 1 Servicio Nacional de Pesca y Acuicultura, Valparaíso, Chile; 2 Veterinary Histology and Pathology, Institute of Animal Health and Food Safety, Veterinary School, University of Las Palmas de Gran Canaria, Las Palmas de Gran Canaria, Spain; 3 Departamento de Medicina Preventiva Animal, Facultad de Ciencias Veterinarias y Pecuarias, Universidad de Chile, Santiago, Chile; 4 Programa de Doctorado en Ciencias Silvoagropecuarias y Veterinarias, Universidad de Chile, Santiago, Chile; 5 Servicio Agrícola y Ganadero, Santiago, Chile; 6 Escuela de Medicina Veterinaria, Facultad de Ciencias de la Vida, Universidad Andrés Bello, Santiago, Chile; 7 Departamento de Patología y Medicina Preventiva, Facultad de Ciencias Veterinarias, Universidad de Concepción, Chillán, Chile; 8 Escuela de Medicina Veterinaria, Facultad de Ciencias Biológicas, Facultad de Medicina y Facultad de Agronomía y Sistemas Naturales, Pontificia Universidad Católica de Chile, Santiago, Chile

**Keywords:** avian influenza virus, cetaceans, dolphins, H5N1 subtype, influenza A virus, highly pathogenic avian influenza, porpoises

## Abstract

Between 2022 and 2023, a significant increase in mortality among marine birds and pinnipeds along the Pacific coasts of Peru and Chile was linked to the highly pathogenic avian influenza virus (HPAIV H5N1). In response, the Chilean Fisheries and Aquaculture Service (SERNAPESCA) initiated a survey to document cetacean strandings and assess the presence of HPAIV H5N1 via molecular analysis of swab samples. In 2023, 205 cetacean stranding events involving 281 individuals were recorded. Of the 27 sampled animals, four, comprising two Burmeister’s porpoises (*Phocoena spinipinnis*) and two Chilean dolphins (*Cephalorhynchus eutropia*) from different locations, tested positive for HPAIV H5N1 in spiracle, rectal and genital swabs. Additionally, a necropsy performed on a long-beaked common dolphin (*Delphinus delphis*), which had tested negative on swabs, revealed the presence of HPAIV H5N1 through PCR analysis of lung and brain tissues. Histopathological examination showed mild, multifocal lymphoplasmacytic-histiocytic and suppurative leptomeningitis, consistent with lesions associated with HPAIV H5N1 infection, although immunohistochemistry was negative. While the virus’s pathogenic role in cetaceans remains inconclusive due to the limited number of necropsies performed, the detection of HPAIV H5N1 in five cetaceans (two porpoises and three dolphins), Four through external swabs —and one dolphin through internal organs—alongside with elevated mortality in birds and pinnipeds, suggests a potential association between cetacean strandings and HPAIV H5N1 outbreaks. This epidemiological finding warrants further investigation.

## 1 Introduction

In December 2022, the Highly Pathogenic Avian Influenza Virus (HPAIV) H5N1 of clade 2.3.4.4b was first identified in Chile, marking the onset of a significant outbreak along the Pacific coasts of Peru and Chile (1–3). This outbreak led to extensive mortalities among avian species and pinnipeds, raising concerns about the virus’s impact on marine ecosystems (4).

While influenza A virus (IAV) infections have been reported in marine mammals, particularly seals and sea lions, occurrences in cetaceans are uncommon but noteworthy. Recent reports of HPAIV H5N1 in cetaceans include cases involving a long-beaked common dolphin (*Delphinus delphis*) from Peru (2), and a harbor porpoise (*Phocoena phocoena*) from Sweden (5). Additionally, a bottlenose dolphin (*Tursiops truncatus*) in Florida exhibited central nervous system (CNS) involvement, with neuronal necrosis and inflammation of the brain and meninges, indicating severe neurological effects of HPAIV H5N1 (6).

Chile has experienced a notable increase in cetacean strandings along its coastline, and a growing concern over the past decade, particularly concerning Unusual Mass Mortality Events (UMEs) in Chilean Central Patagonia (7). Some of the mass strandings have been attributed to natural causes, such as harmful algal blooms (HABs) resulting in biotoxin intoxication (8) and disorientation potentially linked to geomagnetic or topographic anomalies (9–11). In contrast, numerous individual strandings are thought to be related to anthropogenic factors, including vessel collisions and entanglement in fishing or aquaculture gear (11).

Excluding two major events—373 sei whales (*Balaenoptera borealis*) in 2015 and 124 pilot whales (*Globicephala melas*) in 2016 (11), the average annual number of cetacean strandings in Chile between 2014 and 2022 was approximately 18.5 individuals. However, since the emergence of HPAIV H5N1, increased stranding reports among marine mammals have been observed. The present study aims to provide an in-depth analysis of cetacean stranding and mortality events in Chile during 2023, with a focus on exploring potential links to the emergence of HPAIV H5N1.

## 2 Materials and methods

The data on cetacean strandings in 2023 was obtained from SERNAPESCA, including georeferenced locations, the number of stranded individuals, and the species involved. For a subset of the strandings, based on logistical considerations, freshness of the carcasses, and location, SERNAPESCA and Livestock &Agricultural Service of Chile (SAG) personnel collected swab samples from spiracle, genitals, and/or rectum of 27 stranded cetaceans (Supplementary Table S1). Swabs were collected individually from each animal and then pooled in a single tube containing denaturant viral transport media (KaiBiLi™ Extended NTM, The Hague, Netherlands). The molecular diagnostic for generic IAV detection was performed using the VetMAX-Gold AIV Detection Kit (Applied Biosystems™). Further confirmation was attempted by specific TaqMan real-time RT-PCR (rRT-PCR) protocols to detect H5 clade 2.3.4.4, determine the H5 pathotyping (whether it is highly pathogenic or not), and identify the N1 subtype, following NVSL protocols (1732.02, 1767.01 and 1768.01) (4, 12).

Due to restrictions imposed by the ongoing outbreak of HPAIV H5N1 among seabirds and marine mammals along the Chilean coastline, necropsies were prohibited except for public services. However, we performed a necropsy on one long-beaked common dolphin in a necropsy facility at Universidad Andrés Bello, Viña del Mar, Chile, by personnel of SERNAPESCA. During this procedure, tissue samples were collected for molecular diagnosis and histological (Hematoxylin and Eosin, H&E) and immunohistochemical (IHC) studies.

Immunohistochemistry was performed on 3-μm-thick formalin-fixed paraffin-embedded (FFPE) tissue sections from the collected samples, including the lung (two sections), pulmonary lymph node, spleen (two sections), small and large intestine (colon), and brain, using a monoclonal antibody (EBS-I-238; Biologicals Limited, https://biologicals-ltd.com External Link) targeting the nucleoprotein of influenza virus type A regardless of the strain, as previously described (4). The immunostaining protocol included a positive control (brain tissue known to show positive reaction with this specific monoclonal antibody and molecularly confirmed positive for H5N1) and a negative control (the same brain section in which the primary antibody was omitted). In addition to the swab-based molecular diagnostics, rRT-PCR was conducted on four 10 μm-thick FFPE sections obtained from the collected samples. RNA was extracted and deparaffinized using the xylene-alcohol method according to the manufacturer’s recommendations (RNeasy FFPE Kit, Qiagen, Inc., Valencia, CA, USA). Positive short PCR products were purified using a Real Clean spin kit (REAL, Durviz, S. L., Valencia, Spain) for sequencing, and the resulting amplicons were identified through BLAST analysis. Additionally, data analysis was performed using FluSurver (Bioinformatics Institute, A*STAR, https://flusurver.bii.a-star.edu.sg/).

Spatial scan statistics were applied to cetacean mortality events from 2023 and to the wild-bird HPAIV 2023 Chilean outbreak data to identify overlaps between both events. Kulldorff space exploration statistic was performed (13) using SatScan_®_ software (14). The Bernoulli model was used to assess local clusters, considering the binary nature of the data (dead = 1, or alive = 0 individuals) (15). For cluster detection, parameters were fixed by default, and the search was only for high-risk clusters.

## 3 Results

In 2023, 205 stranding events were recorded, involving 281 specimens. Nineteen different stranded species were reported, the most informed animals included long beaked common dolphin (*Delphinus delphis*) (56), Burmeister’s porpoise (*Phocoena spinipinnis*) (40), dusky dolphin (*Lagenorhynchus obscurus*) (33), Chilean dolphin (*Cephalorhynchus eutropia*) (18), bottlenose dolphin (*Tursiops truncatus*) (17), sei whale (*Balaenoptera borealis*) (16), sperm whale (*Physeter macrocephalus*) (8), and most of stranding’s were single events (171), and 34 cases reported between 2 and 9 specimens. Two hundred and seventy-one specimens were found dead, while 10 were reported alive at the time of stranding. However, 8 of the live specimens died shortly after stranding, and 2 were successfully released from entanglement. Most animals (248) presented a normal body condition, and only 33 showed a clear emaciation. Sixty-six specimens were in an advanced state of decay making it impossible to classify them to species level. Fourteen out of 15 administrative coastal regions reported cases, with Arica, the northernmost region of Chile, accounting for the majority (152 cases) (Supplementary Table S3). Considering that the average number of stranded cetaceans per year before 2023 was 18.5, we observed a more than 15-fold increase compared to a typical year.

Twenty-seven cetaceans from 13 species were sampled across 26 distinct locations, spanning 11 administrative regions of Chile. Of these 27 individuals, 4 tested positive for HPAIV H5N1 in swab samples, with Ct values ranging from 17.9 to 25 for generic IAV detection. These included 2 of the 5 Burmeister’s porpoises from the Northern Chile, and 2 of the 5 Chilean dolphins from Southcentral Chile, each one from a different location (Supplementary Table S1; Figure 1). All other sampled animals tested negative, except for the necropsied long-beaked common dolphin, which was positive in tissue samples. From positive swabs, the presence of HPAIV H5N1 was confirmed by real-time RT-PCR. For H5 clade 2.3.4.4, the Ct values ranged from 18 to 27; for H5 pathotyping, Ct values ranged from 21 to 24; for N1 subtyping Ct values ranged from 17 to 30. One case that had tracheal and rectal swabs was separately tested to compare results, obtaining slightly lower Ct values in the rectal (Supplementary Table S2). The overall positivity rate for HPAIV H5N1 among the stranded cetaceans was 18.5%, with approximately 40% positivity observed in both Burmeister’s porpoises and Chilean dolphins.

**Figure 1:**
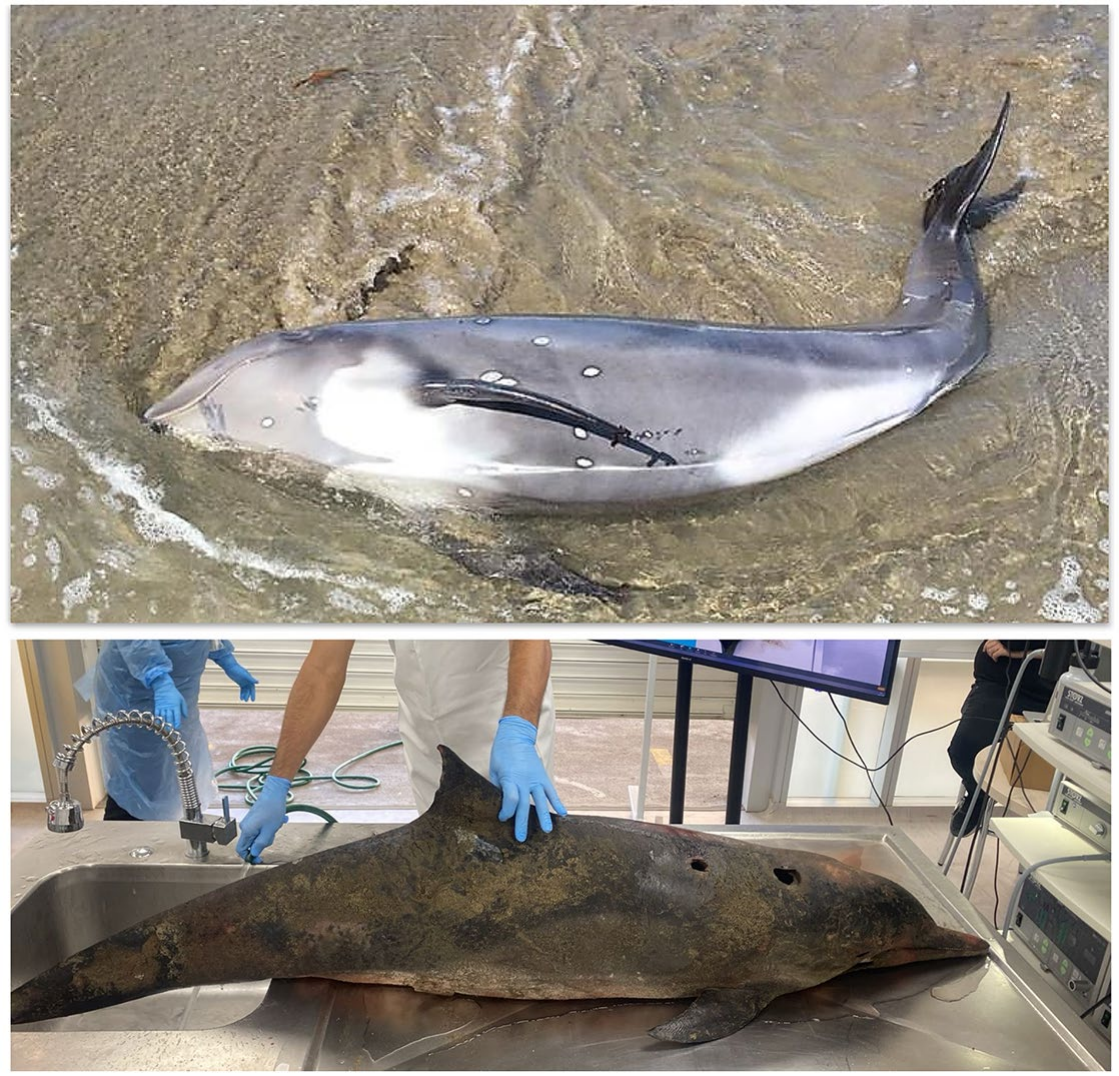
HPAIV H5N1 in a Chilean dolphin (upper) and a necropsied long-beaked common dolphin (*Delphinus delphis*) (down).

The stranded long-beaked common dolphin was initially found alive but died shortly after stranding. The carcass was recovered by the official services 2 days after the initial observation due to logistical limitations. Necropsy revealed moderate putrefaction, signs of trauma associated with the stranding event, such as subcutaneous hemorrhages, and lung congestion and alveolar edema. No parasites or other significant gross abnormalities were observed in the thorax or abdomen. Histopathological examination identified mild leptomeningitis, characterized by lymphoplasmacytic and histiocytic infiltration with occasional neutrophils, suggestive of a chronic immunoinflammatory response. However, the exact cause of death and the stranding event remain inconclusive, as the lesions were not severe enough to cause death (Supplementary information 1). No viral RNA was detected in swab samples from the dolphin. However, PCR analysis yielded positive results in 2 out of 8 tissue samples, specifically from the lung (one section) and brain, with respective cycle threshold (Ct) values of 37 and 29. Sequencing of the small PCR amplicons (about 150 base pairs) (GenBank PQ849341) confirmed the presence of the HPAIV H5 subtype, clade 2.3.4.4b. The consensus sequence was obtained by sequencing both forward and reverse reads and showed 96.05% identity (99% query coverage, QC) with 23 avian sequences from North and South America (Chile, Peru, and Brazil), along with 6 sequences from cattle in North America (Texas), and 95.39% identity (99% QC) with other avian sequences as well as 3 sequences from South American sea lions from Chile and Brazil. Is important to remark that this partial sequence was only obtained to confirm the viral presence and does not represent a detailed genetic analysis.

Despite the positive PCR results, no viral antigen was detected via immunohistochemistry (IHC), potentially reflecting the limitations of IHC in formalin-fixed tissues or the viral load below the detection limit. Microscopic lesions were restricted to the brain, where leptomeningitis was observed, characterized by an infiltration of lymphocytes, plasma cells, macrophages, occasional neutrophils, and leukocytosis (Figure 2). These infiltrates represent a nonspecific immunoinflammatory response, manifesting as leptomeningoencephalitis, typically triggered by pathogens like viruses and bacteria. After ruling out common infectious agents such as Cetacean Morbillivirus (CeMV), *Toxoplasma gondii*, and *Brucella* spp. (Supplementary Table S4), the observed lesions are more likely attributed to infection by HPAIV H5N1, supported by positive H5N1 RT-PCR results. The associated encephalitis may also explain the live stranding and suggest the presence of potential clinical neurological symptoms (Supplementary information, Video).

**Figure 2:**
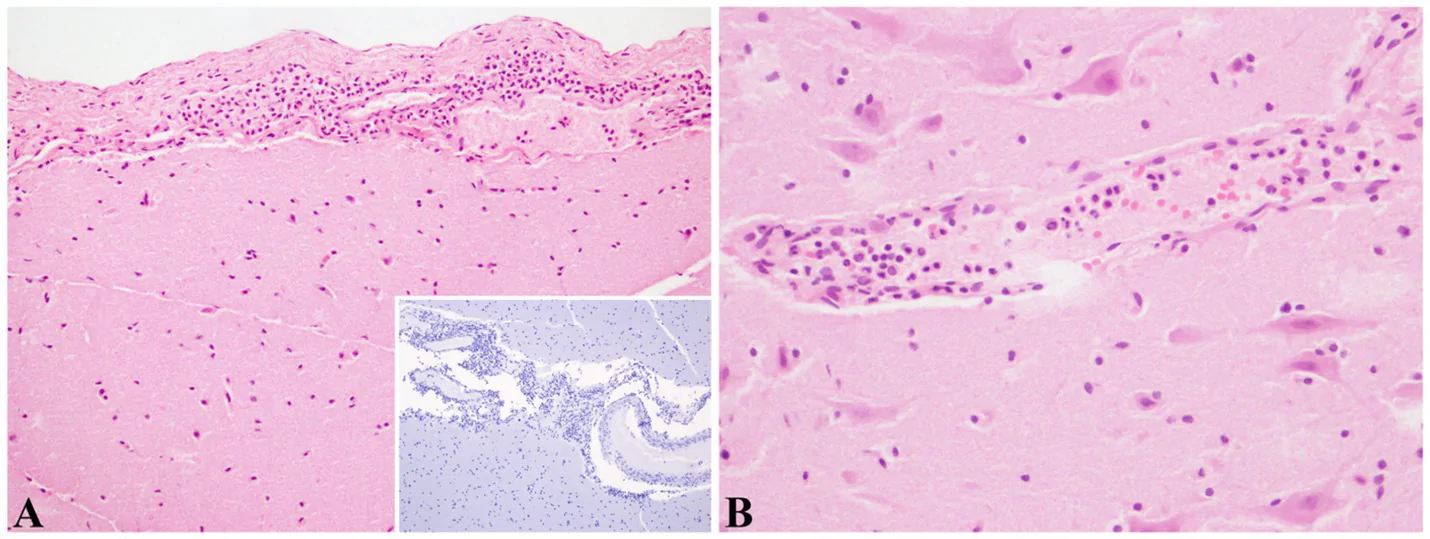
Histopathology from *Delphinus delphis*. **(A)** Mild multifocal lymphoplasma-histiocytic and suppurative leptomeningitis. H&E, 20×. Inset: no viral antigen was detected in association with the inflammatory infiltrate present in the leptomeninges. IHC against influenza A nucleoprotein, 10×. **(B)** Leukocytosis. H&E, 40×.

Finally, the spatial exploration shows two non-statistically significant (*p* > 0.05) high-risk clusters (Supplementary Table S5) located in northern Chile. It also shows a spatial overlap of both stranded cetacean events and HPAIV-positive wild birds during the same time and high-risk clusters, suggesting a spatial association between both events (Figure 3).

**Figure 3:**
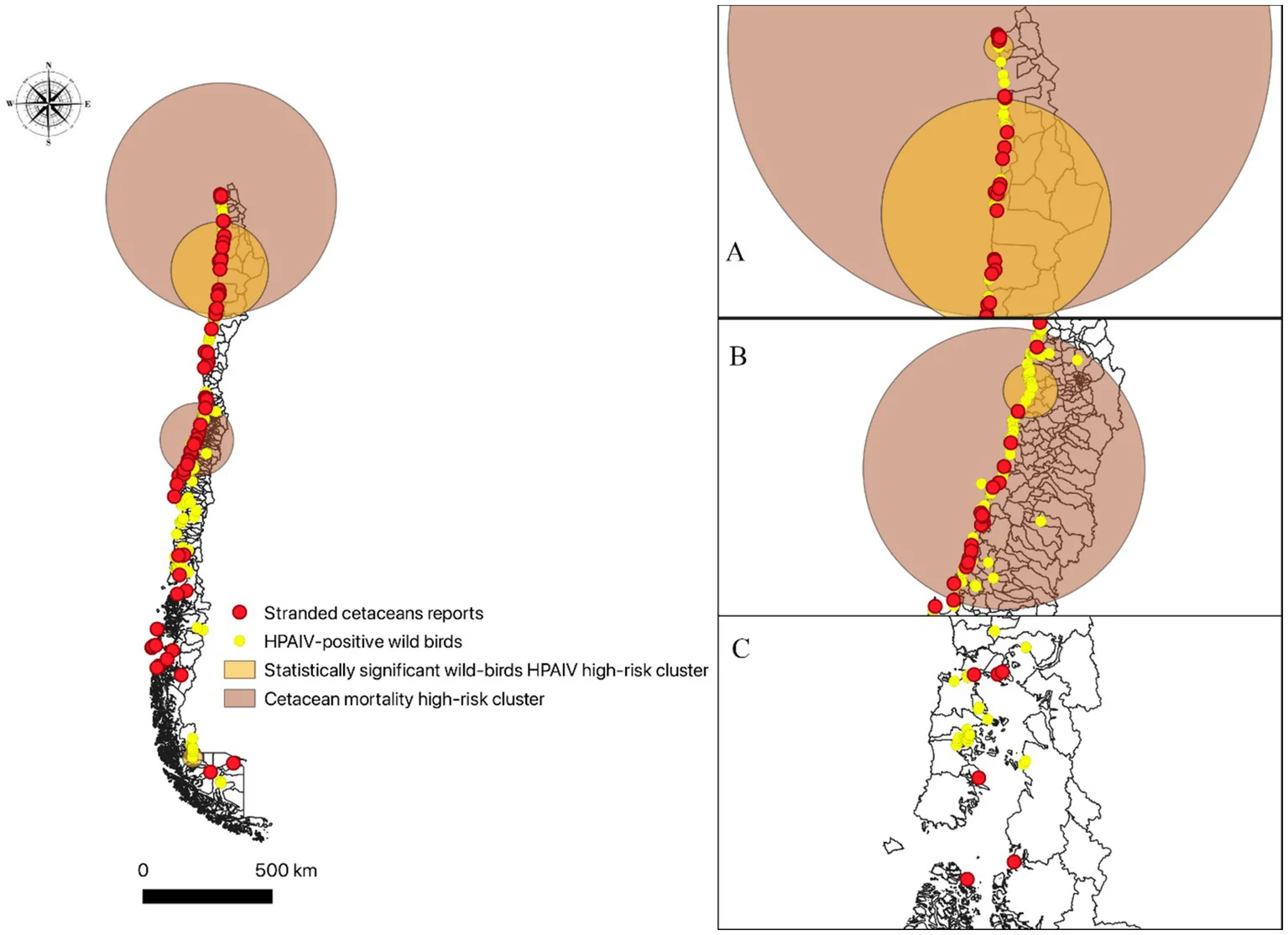
Spatial distribution of stranded cetacean events and HPAIV-positive wild birds in the context of the 2023 Chilean outbreak. **(A)** Spatial overlap of stranded cetaceans reports and HPAIV-positive wild birds, and a high-risk cluster for both mortality of stranded cetaceans reports and HPAIV in wild birds in northern Chile. **(B)** Spatial overlap of stranded cetaceans reports and HPAIV-positive wild birds, and a high-risk cluster for both mortality of stranded cetaceans reports and HPAIV in wild birds in central and part of southern Chile. **(C)** Spatial proximity between stranded cetaceans’ events and HPAIV-positive wild birds in southern Chile.

## 4 Discussion

To date, only three cases of HPAIV H5N1 have been reported in cetaceans, one bottlenose dolphin from Florida (6), one long-beaked common dolphin from Peru (2), and one Harbor Porpoise, from Sweden (5). This report confirms the first detection of HPAIV H5N1 in Burmeister’s porpoises and Chilean dolphins, ratifying that HPAIV H5N1 can infect these species and suggesting the potential to infect other members of the Cetacean order.

In our study and previous reports of HPAIV H5N1, the most likely source of infections are the seabirds, which tested positive in high numbers during the outbreaks. A plausible explanation for the infection source is environmental contamination from surface feeding, sharing habitats with infected seabirds (16, 17). Notably, the increase in cetacean strandings along Chile’s coastline in the first half of 2023 coincided temporally and geographically with outbreaks of HPAIV in sea birds, indicating a significant correlation between HPAIV presence in wild birds and the elevated risk to other species. Similar findings have been reported in Humboldt penguins (12) and South American sea lions (4) during the same period and in the same regions.

Evidence of IAV infection in cetaceans has historically been scarce, with only three preceding reports. The first involved the isolation of the H1N3 subtype from the lungs and liver of a striped whale (Balaenopteridae) collected by scientists in a whaling boat in the South Pacific Ocean in 1975/1976 (18). The second one described the isolation of H13N2 and H13N9 subtypes from pilot whales (*Globicephala melas*) during two mass stranding events in in New England, Maine, USA in 1984. These events involved 97 and 23 specimens in October and November, respectively (19). In all cases, the viruses were genetically related to those isolated in seabirds (20). In contrast, although there have been significant stranding events involving sei whales (16), especially in Chile’s Northern Patagonia, these cases are often linked to biotoxin intoxication, particularly in the Aysen region, where mass mortality of baleen whales due to harmful algal blooms (HAB) has been suggested (11).

Summarizing, our study highlights a remarkable surge in cetacean strandings along Chile’s coastline in 2023, which correlates both geographically and temporally with the HPAIV H5N1 outbreak in birds and sea lions, suggesting a possible connection. We confirmed the presence of HPAIV H5N1, clade 2.3.4.4b, in Burmeister’s porpoises and Chilean dolphins through rRT-PCR testing of swabs from external orifices. In contrast, evidence of infection in a long-beaked common dolphin was identified via necropsy, histopathological analysis, and rRT-PCR testing of internal organs, although IHC failed to detect the virus. Microscopic examination of brain lesions in the common dolphin revealed features similar to those previously described in cases of H5N1 HPAIV infection in birds and terrestrial mammals from the Gs/Gd lineage. However, key pathological markers such as perivascular cuffing and neuronal necrosis in the grey matter of the cerebral hemispheres, commonly associated with AIV infection (21), were absent. This absence may explain the negative IHC results in our study, similar to findings in other investigations (22, 23), where neurons and glial cells linked to such lesions typically express viral proteins when infected with HPAIV in the CNS (21), It is also possible that more severe lesions were present in unsampled regions of the CNS, given that video evidence of the dolphin’s stranding event suggested neurological involvement before death. Moreover, the potential contribution of other pathogens typically associated with these brain lesions in cetaceans (24) was ruled out through molecular testing. Also, the lack of IHC-positive samples in a confirmed and sequence animal could be explained by differences in sensitivity between the techniques (22).

We also acknowledge several limitations in this study. One major limitation was the low proportion of stranded animals that were sampled, primarily due to a shortage of personnel and the overwhelming situation during the first half of 2023, when thousands of seabirds were found dead along the shore. Additionally, the prioritization of sampling cases with higher human health risks, such as outbreaks in backyard farms, further reduced the number of sampled cetaceans. Importantly, during this period, government authorities restricted access to stranding sites for NGOs and other institutions due to concerns over the zoonotic potential of HPAIV H5N1, which also limited sampling efforts. Considering these limitations, sampling was not opportunistic in nature; rather, the low sampling coverage was a direct consequence of the lack of available personnel and institutional restrictions. This experience highlights the importance of revisiting future outbreak response policies to enable the safe and coordinated participation of NGOs and academic institutions, which could significantly enhance data collection and the understanding of emerging zoonotic threats in wildlife, as was observed in Perú (3).

Despite these challenges, an 18.5% positivity rate was observed among the sampled animals, which is comparable to the rate reported in South American sea lions (20%) (4) and higher than that observed in Humboldt penguins (2%) (12) or the overall results during the 2022–2023 HPAIV outbreak in Chile (8.5%) (25). The number of RT-PCR-negative animals can be attributed to factors such as the type of sample collected, environmental conditions, and the virus’s pathogenesis (4). In the context of an HPAIV H5N1 outbreak causing widespread mortality, the likelihood of other pathogens being responsible for the observed deaths is expected to be low. Another limitation is that the histopathological analysis could not confirm an association between H5N1 infection and neurological signs in this study. Finally, it is important to emphasize that this study was not designed to genetically characterize the virus in cetaceans but rather to provide a comprehensive overview of the epidemiological context and its implications for cetacean strandings during the HPAIV H5N1 outbreak in the Chilean coast during 2023.

This discovery is significant, as only three previous cases of HPAIV H5N1 infection have been reported in cetaceans worldwide. Given the potential for a HPAIV H5N1 virus outbreak in cetacean species, it is crucial to investigate and understand the factors involved in these incidents. Moreover, as whales are highly migratory species, moving between tropical and polar regions, being at increased risk of collisions with vessels, entanglements in marine debris or fishing gear, and acoustic contamination, all of which contribute to mortality, a combined such set of circumstances with the potential threat of HPAIV, could pose a significant challenge to the survival of cetacean populations, as has already been observed in other marine mammal species in Chile.

This study aimed to investigate cetacean strandings and mortalities that occurred during the HPAIV H5N1 epizootic event, and based on available data, to discuss potential transmission routes of the virus within and beyond cetacean species.

## Data Availability

The original contributions presented in the study are included in the article/Supplementary material, further inquiries can be directed to the corresponding authors.
