# Supplementary material for: Highly pathogenic avian influenza virus H5N1 infection in stranded porpoises and dolphins in Chile
Source: Front Vet Sci. 2026 Oct 20;13:1969182. doi: 10.3389/fvets.2026.1969182 (PMC13729797; doi:10.3389/fvets.2026.1969182)
Supplement: Supplementary file 1 [file Table_1.docx]

Supplementary Material

Supplementary Table 1. Sample collection of stranded cetaceans arranged by date.

| **Id** | **Region** | **Location** | **Date** | **Common Name** | **Result** |
| --- | --- | --- | --- | --- | --- |
| 1 | Coquimbo | Playa Peñuelas | 02-22-2023 | Burmeister's porpoise | Negative |
| 2 | Maule | Sector Costero | 03-04-23 | Humpback whale | Negative |
| 3 | Maule | Chanco | 03-18-2024 | Chilean dolphin | Negative |
| 4 | Maule | Playa Iloca | 03-18-2024 | Chilean dolphin | Negative |
| 5 | O'Higgins | Pichilemu | 03-19-2023 | Cuvier's beaked whale | Negative |
| 6 | Atacama | Playa Las Lisas | 03-27-2023 | Burmeister's porpoise | Positive (pool Ct:19.3) |
| 7 | Maule | Caleta Duao | 03-31-2023 | Chilean dolphin | Positive (pool Ct:24.8) |
| 8 | Antofagasta | Playa Punta Itata | 03-31-2023 | Burmeister's porpoise | Positive (spiracle, Ct17.9; oral, Ct 20.7) |
| 9 | O'Higgins | Playa Matanzas | 04-09-23 | Burmeister's porpoise | Negative |
| 10 | Ñuble | Playa Punta Mela | 04-01-23 | Chilean dolphin | Positive (spiracle, Ct 25, rectal Ct, 23. |
| 11 | Valparaiso | Playa La Ballena | 04-20-2023 | Risso's dolphin | Negative |
| 12 | Atacama | Chañaral | 04-24-2023 | Dwarf sperm whale | Negative |
| 13 | Tarapaca | Peninsula Iquique | 04-29-2023 | Rough-toothed dolphin | Negative |
| 14 | Valparaiso | Papudo | 04-27-2023 | Long-beaked common dolphin | Negative to swabs. Positive tissues |
| 15 | Tarapaca | Peninsula Iquique | 05-08-23 | Dusky dolphin | Negative |
| 16 | Ñuble | Coelemu | 06-06-23 | Chilean dolphin | Negative |
| 17 | Biobio | Lebu | 06-01-23 | Risso's dolphin | Negative |
| 18 | Atacama | Copiapo | 06-04-23 | Sperm whale | Negative |
| 19 | Aysen | Pto Cisnes | 06-25-2023 | Southern right whale | Negative |
| 20 | Coquimbo | La Serena | 07-03-23 | Burmeister's porpoise | Negative |
| 21 | Coquimbo | Playa Los Choros | 07-10-23 | Bottlenose dolphin | Negative |
| 22 | Los Lagos | Puerto Elvira-Ancud | 08-05-23 | Fin whale | Negative |
| 23 | Coquimbo | Las Salinas-Canela | 09-02-23 | Fin whale | Negative |
| 24 | Los Lagos | Quenuir-Maullin | 11-10-23 | Sperm whale | Negative |
| 25 | Antofagasta | La Rinconada | 11-12-23 | Long-beaked common dolphin | Negative |
| 26 | Biobío | Lebu | 11-24-2023 | Long-beaked common dolphin | Negative |
| 27 | Coquimbo | La Higuera | 12-11-23 | Dwarf sperm whale | Negative |

Supplementary Table 2. Subtyping of positive samples.

| **Id** | **Common Name** | **H5 clade 2.3.4.4** | **H5 pathotyping** | **N1 subtyping** |
| --- | --- | --- | --- | --- |
| 6 | Burmeister's porpoise | 18 | 22 | 17 |
| 7 | Chilean dolphin | 27 | 23 | 29 |
| 8 | Burmeister's porpoise | 24 | Not tested | Not tested |
| 10 | Chilean dolphin (tracheal) | 23 | 24 | 30 |
| 10 | Chilean dolphin (rectal) | 20 | 21 | 28 |

| **Supplementary table 3. Number of stranded cetaceans by species and regions, 2023** | **NORTHERN** | | | | | **CENTRAL** | | **SOUTH CENTRAL** | | | | **SOUTHERN** | | | |  |
| --- | --- | --- | --- | --- | --- | --- | --- | --- | --- | --- | --- | --- | --- | --- | --- | --- |
| **Species/Regions** | **Arica** | **Tarapacá** | **Antofagasta** | **Atacama** | **Coquimbo** | **Valparaiso** | **O’Higgins** | **Maule** | **Ñuble** | **Biobío** | **Araucanía** | **Los Ríos** | **Los Lagos** | **Aysen** | **Magallanes** | **TOTAL** |
| Burmeister's porpoise (*Phocoena spinipinnis*) | 21 |  | 5 | 2 | 7 | 2 | 1 | 1 |  | 1 |  |  |  |  |  | 40 |
| Chilean dolphin (*Cephalorhynchus eutropia*) |  |  |  |  |  |  |  | 8 | 6 |  |  |  | 3 |  | 1 | 18 |
| Sei whale (*Balaenoptera borealis*) |  |  |  |  |  |  |  |  |  |  |  |  |  | 16 |  | 16 |
| Dusky dolphin (*Lagenorhynchus obscurus*) | 17 | 9 | 4 |  | 4 |  |  |  |  |  |  |  |  |  |  | 34 |
| Bottlenose dolphin (*Tursiops truncatus*) | 14 |  |  |  | 1 | 1 | 1 |  |  |  |  |  |  |  |  | 17 |
| Common dolphin (*Delphinus delphis*) | 48 |  | 4 | 1 | 1 | 1 |  | 1 |  |  |  |  |  |  |  | 56 |
| Humpback whale (*Megaptera novaeangliae*) |  |  |  | 2 |  |  |  | 1 |  | 2 |  | 1 | 1 |  |  | 7 |
| Risso's dolphin (*Grampus griseus*) |  |  |  |  | 1 | 1 |  |  |  | 1 |  |  |  |  |  | 3 |
| Killer whale (*Orcinus orca*) |  |  |  |  |  |  |  |  |  | 1 |  |  |  |  | 1 | 2 |
| Fin whale (*Balaenoptera physalus)* |  |  |  |  | 1 |  |  | 1 |  | 1 |  |  | 1 |  |  | 4 |
| Couvier´s beaked whale (*Ziphius cavirostris*) |  |  |  |  |  |  | 1 |  |  |  |  |  |  |  |  | 1 |
| Pygmy sperm whale (*Kogia breviceps*) |  |  |  | 1 | 1 |  |  |  |  |  |  |  |  |  |  | 2 |
| Sperm whale - (*Physeter macrocephalu*s) |  | 2 | 1 | 1 | 1 |  |  |  |  | 2 |  |  | 1 |  |  | 8 |
| Rough-toothed dolphin (*Steno bredanensis*) |  | 1 |  |  |  |  |  |  |  |  |  |  |  |  |  | 1 |
| Arnoux's beaked whale (*Berardius arnuxii*) |  |  |  |  |  |  |  |  |  |  |  |  |  |  | 1 | 1 |
| Southern right whale-(*Eubalena australis)* |  |  |  |  |  |  |  |  |  |  |  |  |  | 1 |  | 1 |
| Layard´s beaked (*Mesoplodon layardi*) |  |  |  |  | 1 |  |  |  |  |  |  |  |  |  |  | 1 |
| Peale´s dolphin (Lagenorhynchus australis) |  |  |  |  |  |  |  |  |  |  |  |  |  | 1 |  | 1 |
| Spinner dolphin (*Stenella longirostris*) |  |  |  |  |  |  |  |  |  | 2 |  |  |  |  |  | 2 |
| Undetermined | 52 | 3 | 3 | 1 | 2 | 1 | 2 | 1 |  |  |  |  | 1 |  |  | 66 |
| TOTAL | 152 | 15 | 17 | 8 | 20 | 6 | 5 | 13 | 6 | 10 | 0 | 1 | 7 | 18 | 3 | 281 |

Supplementary Table 4. RT-PCR and IHC for the necropsied *Delphinus delphis.*

| **Tissue** | **RESULTS** | | |
| --- | --- | --- | --- |
|  | **PCR AIV** | **IHC AIV** | **CeMV, *T. gondii***  ***Brucella* spp.** |
| Pulmonary Lymph Node | - | - | - |
| Spleen | - | - | - |
| Spleen | - | - | - |
| CNS | + | - | - |
| Lung | + | - | - |
| Lung | - | - | - |
| Small Intestine | - | - | - |
| Colon | - | - | - |

Supplementary Table 5. High-risk clusters for mortality of cetaceans during Chilean HPAIV outbreak in 2023.

| **Cluster** | **Latitude** | **Longitude** | **Radius (km)** | **Relative risk** | **p-value** |
| --- | --- | --- | --- | --- | --- |
| 1 | -18.4424 | -70.3054 | 922.79 | 1.09 | 0.154 |
| 2 | -34.8833 | -72.1612 | 254.59 | 1.06 | 0.997 |

# Supplementary information 1.

## 1.1 Necropsy summary:

Live stranded dolphin which died and was recovered from the beach a day after, and it was frozen until this thawed dolphin carcass was used for necropsy capacity building. The body showed a moderate to poor body condition with a moderate putrefactive stage when necropsy was performed. Externally, several rounded skin and blubber areas in the thorax were preyed on by scavenging birds. During necropsy, focal subcutaneous hemorrhages were found as well as blood within the scapula-humeral joints. Both injuries were due to trauma during live stranding. Encapsulated subcutaneous abscess in the neck (5cm) not associated anatomically with the trachea or esophagus. Overinflated congestive lungs with alveolar edema in the trachea and no parasites in the respiratory tract were the main gross findings in the thoracic cavity. Heart without macroscopic lesions and without coronary fat. And no stomach content as well as no gross lesions in the proximal and distal intestine (without parasites) were the abdominal cavity gross findings. No macroscopic lesions and no parasites in the liver. No enlarged lymph nodes were detected in the thoracic or abdominal cavity. The whole brain was recovered for histological analysis. No gross lesions were observed externally or internally in this organ.

Conclusion: The cause of dead as well as cause of stranding remains unknown. The necropsy did not show any significant gross finding that could be considered linked to disease and or death. Microscopically, leptomeningitis could histopathologically be of concern regarding previous dolphin health and or be associated with a live stranding case, but the final etiology of that chronic inmuno-inflammatory reaction was not possible to be identified.

Supplementary information, Video. Stranded Long-beaked common dolphin, before necropsy.
